# Canine mesenchymal stem cells from synovium have a higher chondrogenic potential than those from infrapatellar fat pad, adipose tissue, and bone marrow

**DOI:** 10.1371/journal.pone.0202922

**Published:** 2018-08-23

**Authors:** Akari Sasaki, Mitsuru Mizuno, Nobutake Ozeki, Hisako Katano, Koji Otabe, Kunikazu Tsuji, Hideyuki Koga, Manabu Mochizuki, Ichiro Sekiya

**Affiliations:** 1 Center for Stem Cell and Regenerative Medicine, Tokyo Medical and Dental University, Tokyo, Japan; 2 Department of Veterinary Medical Sciences, the University of Tokyo, Tokyo, Japan; 3 Department of Cartilage Regeneration, Tokyo Medical and Dental University, Tokyo, Japan; 4 Department of Joint Surgery and Sports Medicine, Tokyo Medical and Dental University, Tokyo, Japan; Augusta University, UNITED STATES

## Abstract

Osteoarthritis (OA), a common chronic joint disorder in both humans and canines, is characterized by a progressive loss of articular cartilage. Canines can serve as an animal model of OA for human medicine, and this research can simultaneously establish effective veterinary treatments for canine OA. One attractive treatment that can lead to cartilage regeneration is the use of mesenchymal stem cells (MSCs). However, for canine OA, little information is available regarding the best source of MSCs. The purpose of this study was to identify a promising MSC source for canine cartilage regeneration. We collected synovial, infrapatellar fat pad, inguinal adipose, and bone marrow tissues from six canines and then conducted a donor-matched comparison of the properties of MSCs derived from these four tissues. We examined the surface epitope expression, proliferation capacity, and trilineage differentiation potential of all four populations. Adherent cells derived from all four tissue sources exhibited positivity for CD90 and CD44 and negativity for CD45 and CD11b. The positive rate for CD90 was higher for synovium-derived than for adipose-derived and bone marrow-derived MSCs. Synovium-derived and infrapatellar fat pad-derived MSCs displayed substantial proliferation ability, and all four populations underwent trilineage differentiation. During chondrogenesis, the wet weight was heavier for cartilage pellets derived from synovium MSCs than from the other three sources. The synovium is therefore a promising source for MSCs for canine cartilage regeneration. Our findings provide useful information about canine MSCs that may be applicable to regenerative medicine for treatment of OA.

## Introduction

Osteoarthritis (OA), the most common chronic disorder of synovial joints, is characterized by the progressive loss of articular cartilage, which leads to pain and functional impairment. Current treatment options are limited to analgesia and to prosthetic joint replacement for end-stage disease. An unmet need still exists for the development of regenerative medicine that can restore lost cartilage and thereby provide a long-term solution for OA symptoms. [1, 2]

OA is not specifically a human disease, as canines can spontaneously develop OA, and this is becoming a significant veterinary problem in aging companion dogs [3–5]. For this reason, the development of regenerative medicine for OA is anticipated in veterinary medicine as well as in human medicine. In this context, clinical studies carried out on canines may have significant value in establishing the safety and efficacy of regenerative medicine for OA treatments in humans, because long-term follow-up is possible in companion dogs.

One attractive regenerative approach for cartilage regeneration is the use of mesenchymal stem cells (MSCs), which can be isolated from various mesenchymal tissues of both dogs and humans. Most canine studies use MSCs derived from bone marrow or subcutaneous adipose tissue [6–11], whereas synovium or infrapatellar fat pad tissues have been viewed as promising MSC sources for cartilage regeneration in other animal species [12–18]. However, few studies have characterized the MSCs derived from canine synovium or infrapatellar fat pad, particularly in terms of their chondrogenic capacity. Previous canine studies have also used different processes for MSC expansion, which precludes direct comparison of canine MSCs from different sources, as their properties can be affected by the preparation methods. Therefore, eligible MSC sources for canine cartilage regeneration remain to be clarified.

In the present study, we expanded MSCs from different canine tissues using strictly controlled and similar processes, and we performed a donor-matched quantitative comparison of the MSC properties. Our results show that canine MSCs isolated from synovium, infrapatellar fat pad, adipose, and bone marrow tissues exhibit similarities and differences, and the data provide useful information on canine MSCs that may be applicable to regenerative medicine for OA.

## Materials & methods

### Tissue collection from canines

**Fig 1:**
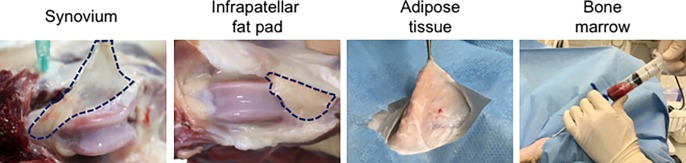
Tissue collection. Synovium and the infrapatellar fat pad were harvested from the knee joint, adipose tissue from the inguinal region, and bone marrow from the humerus.

Six healthy, skeletally mature beagle dogs (12–18 months old, 10–17 kg, 5 males and 1 female) were used in the study. All experiments were conducted in accordance with our institutional guidelines. The protocol was approved by the Animal Committee of Tokyo Medical and Dental University (Protocol number: 0170403A) and the Animal Committee of the Graduate School of Agricultural and Life Sciences at the University of Tokyo (Protocol number: P16-279). Dogs were euthanized with an overdose intravenous injection of thiopental (150 mg/kg) or deep anesthesia with isoflurane followed by an intravenous KCL injection (1 ml/kg) for reasons unrelated to this study, including an odontological study (approved by the Animal Committee of Tokyo Medical and Dental University; approval number: 0170333A) and ophthalmological studies (the Animal Care Committee of the Graduate School of Agricultural and Life Sciences at the University of Tokyo; approval number: P15-13, PH15-81 and PH17-116). After euthanasia, synovium with subsynovial tissue was harvested from each dog from the bony side of the suprapatellar pouch in the knee. The infrapatellar fat pad was harvested from the knee joint. Subcutaneous adipose tissue was harvested from the inguinal region. Bone marrow was aspirated from the proximal humerus using bone marrow puncture needles (Fig 1).

### Preparation of cells

Synovium, infrapatellar fat pad, and adipose tissues were minced and digested at 37°C for 3 hours in a 3 mg/ml collagenase D solution (Roche Diagnostics, Mannheim, Germany) in α-minimal essential medium (αMEM; Invitrogen, Carlsbad, CA, USA). The cells released by digestion were filtered through a 70-μm nylon filter (Becton Dickinson, Franklin Lakes, N.J., USA), and the remaining tissues were discarded. The obtained nucleated cell numbers were counted for all tissues using a Luna-FL Dual Fluorescence Cell Counter (Logos Biosystems, Annandale, VA, USA). The cells were plated at 1 × 10^3^, 1 × 10^4^, or 1 × 10^5^ cells/dish in 6 dishes for each density (60 cm^2^ culture dish; Nalge Nunc International, Rochester, N.Y., USA) and cultured in complete culture medium: αMEM containing 10% fetal bovine serum (FBS; Invitrogen), 100 units/ml penicillin (Invitrogen), 100 μg/ml streptomycin (Invitrogen), and 250 ng/ml amphotericin B (Invitrogen). Bone marrow was diluted with αMEM and the number of nucleated cells was counted with a Luna-FL Dual Fluorescence Cell Counter. Then bone marrow-derived cells were plated at 1 × 10^4^, 1 × 10^5^, or 1 × 10^6^ cells/60 cm^2^ in complete culture medium. The plated cells were incubated at 37°C in a 5% humidified CO_2_ atmosphere as passage-0. The medium was changed every 3–4 days.

After 14 days, three dishes for each cell concentration were fixed with 10% formalin solution (Wako, Osaka, Japan), stained with 0.5% crystal violet for 5 min, and washed with distilled water. The optimal initial cell density was determined based on the following observations: 1) the colony size was not affected by contact inhibition, and 2) the greatest number of colonies was obtained. We harvested the cells from the remaining 3 dishes and counted them to determine the cell number at passage-0. The data were analyzed with the Friedman test, followed by Dunn’s multiple comparisons test. The passage-0 cells were then replated at 3000 cells/dish (60 cm^2^ dishes) as passage-1 cells and cultured for 14 days. Surface epitopes were then analyzed in 1 × 10^6^ passage-1 cells, while the remaining cells were cryopreserved at a concentration of 1 × 10^6^ cells/ml in αMEM containing 5% dimethylsulfoxide (Wako, Osaka, Japan) and 10% FBS. Aliquots of 1 ml were slowly frozen and cryopreserved at -80°C in a BICELL biofreezing vessel (Nihon Freezer, Tokyo, Japan). The cells were later expanded by thawing a frozen vial of cells, plating them at 3 × 10^5^ cells/dish (150-cm^2^ dishes), and incubating for 7 days in the recovery plate. These passage-2 cells were used in subsequent analyses.

### Colony-forming ability and proliferation potential

The colony-forming abilities of the mesenchymal tissue-derived cell types from 6 canine donors were examined by replating passage-2 cells at 1 × 10^3^ cells/dish (60 cm^2^ dishes) in 6 dishes and incubating them for 14 days in complete medium. Three dishes were then fixed with 10% formalin solution, stained with 0.5% crystal violet (CV), and washed with distilled water for counting of colony numbers in the dishes. Colonies less than 2 mm in diameter or colonies with only faint staining were ignored. The cells from the remaining three dishes were harvested and counted with a hemocytometer to calculate the fold increase and the cell number per colony for each population. The data were analyzed with the Kruskal Wallis test, followed by Dunn’s multiple comparisons test.

Proliferation potentials were compared by plating cells derived from the four canine mesenchymal tissues at passage-2. Cells were plated at 1 × 10^3^ cells/10 cm^2^ (3 canine donors, n = 3 in each donor). After 4, 8, and 12 days of culture, the cells were harvested and the cell numbers were counted. The data were analyzed with the Friedman test, followed by Dunn’s multiple comparisons test.

### Flow cytometry

Passage-1 cells (1 × 10^6^ cells) were suspended in 100 μl phosphate buffered saline (PBS) containing 10 μg/ml antibodies for APC-conjugated CD90 (Becton Dickinson, Franklin Lakes, NJ, USA), PE-conjugated CD44 (eBioscience, San Diego, CA, USA), FITC-conjugated CD45 (eBioscience), and PerCP-Cyanine5.5-conjugated CD11b (Biolegend, San Diego, CA, USA). As an isotype control, nonspecific anti-mouse IgG coupled with APC, PE, FITC, or PerCP-Cyanine5.5 (Becton Dickinson) was substituted for the primary antibody. After incubation for 30 minutes at 4°C, the cells were washed with PBS and then resuspended in 500 μl of PBS for analysis. Cell fluorescence was evaluated by flow cytometry using a FACSVerse instrument (Becton Dickinson); the data were analyzed using Flowjo software (Tree Star, Ashland, OR, USA). All analyses were performed on samples from all 6 donors. The data were analyzed with the Kruskal Wallis test, followed by Dunn’s multiple comparisons test.

### Chondrogenesis

Passage-2 cells (2.5 × 10^5^ cells) for chondrocyte differentiation were placed in a 15-ml polypropylene tube (Falcon, Bedford, MA, USA) and centrifuged at 490 *g* for 10 minutes. The pellets were then cultured for 21 days in a chondrogenic medium containing 250 ng/ml bone morphogenic protein 2 (BMP-2; Medtronic, TN, USA), in high-glucose Dulbecco’s modified Eagle’s medium (Invitrogen) supplemented with 10 ng/ml transforming growth factor-β (TGFβ; Miltenyi Biotec K.K., Tokyo, Japan), 10^−7^ M dexamethasone (Sigma-Aldrich, St. Louis, MO, USA), 50 μg/ml ascorbate-2-phosphate, 40 μg/ml proline, 100 μg/ml pyruvate, and 50 mg/ml ITS+TMPremix (Becton Dickinson). Control pellets were cultured in a similar medium but without supplementation with TGFβ or BMP-2. The medium was changed every 3–4 days. After 21 days, cartilage pellets were photographed and then their wet weight and diameter were measured. The data were analyzed with the Kruskal Wallis test, followed by Dunn’s multiple comparisons test.

For histological analysis, the pellets were embedded in paraffin, cut into 5-μm sections, and stained with toluidine blue. Collagen type II expression was visualized by immunostaining using a rabbit anti-collagen type II antibody (Cosmo Bio Co., LTD., Tokyo, Japan).

### Sulfated glycosaminoglycan (sGAG) and DNA of cartilage pellets

After 21 days of culture, pellets from each tissue were digested for 18 hours at 65°C in papain buffer (200 mM sodium phosphate buffer, 100 mM sodium acetate, 10 mM ethylenediaminetetraacetic acid (EDTA), 5 mM L-cysteine HCl, pH 6.4) containing 5 μg/ml of papain and then centrifuged at 10000*g* for 10 minutes. The sulfated glycosaminoglycan (sGAG) concentration in the supernatant was determined with the Blyscan GAG assay (Biocolor Ltd, Newtonabby, Ireland) according to the manufacturer’s instructions. The total DNA content of the supernatant was quantified using the PicoGreen dsDNA assay kit (Molecular Probes, Eugene, OR, USA) according to the manufacturer’s instructions. The level of sGAG was normalized against the total amount of DNA. The data were analyzed with the Friedman test, followed by Dunn’s multiple comparisons test.

The amounts of DNA in the pellets at day 0 were determined by pelleting 2.5 × 10^5^ passage-2 cells from 3 canine donors, 3 pellets each, as already described mentioned. DNA was immediately extracted from the pellets using the NucleoSpin Extract kit (Machery‐Nagel, Düren, Germany), and the amount of DNA in each pellet was determined. The data were analyzed with the Friedman test, followed by Dunn’s multiple comparisons test.

### Calcification

Passage-2 cells from each tissue were plated at 1 × 10^3^ cells per well in 6-well plates (Falcon) and cultured in complete medium for 7 days (3 canine donors, 3 wells each). The medium was then switched to a calcification medium consisting of complete medium supplemented with 10 mM β-glycerophosphate (Sigma-Aldrich), 100 nM dexamethasone (Sigma-Aldrich), and 50 μM ascorbic acid (Sigma-Aldrich). Control cells were cultured in complete medium without any of these three supplements. After 21 days, the calcification and the control cultures were fixed in 10% formalin solution for 5 minutes, stained with 0.5% alizarin red solution for 5 minutes, and washed with distilled water.

For quantification, 1 × 10^3^ cells were plated in 6-well plates and cultured for 7 days. The medium was then switched to the calcification medium for an additional 21 days. After staining with alizarin red, the areas positive for alizarin red were measured with Image J software [19]. The dishes were then stained with crystal violet and the positively stained areas was measured. The ratio of alizarin red positive area/crystal violet positive area (%) was then calculated. The data were analyzed with the Kruskal Wallis test, followed by Dunn’s multiple comparisons test.

### Adipogenesis

Passage-2 cells from each tissue were plated at 2 × 10^4^ cells/well in 12-well plates (Falcon) (3 canine donors, 3 wells each) and cultured in complete medium for 7 days. The culture medium was then switched to an adipogenic medium consisting of complete medium supplemented with 1 μM dexamethasone (Sigma-Aldrich), 5 μM rosiglitazone (Wako), 5 μg/ml insulin (Wako), and 3.5 g/L glucose (Sigma-Aldrich) [9]. Control cells were cultured in the complete medium without any supplements. After 21 days, the adipogenic and control cultures were fixed in 10% formalin solution for 1 hour, stained with fresh oil red-o solution for 2 hours, and then washed with distilled water. The oil red-o solution was prepared by mixing three parts of stock solution (0.5% in isopropanol; Sigma-Aldrich) with two parts of Milli-Q water and filtering through a 0.45-μm filter.

For quantification, nine areas surround by a square 548 μm in height and 730 μm in width were randomly selected from each well, and the ratio of oil-red-o positive areas per total area was measured with Image J software. The data were analyzed with the Kruskal Wallis test, followed by Dunn’s multiple comparisons test.

### Statistical analysis

All data were statistically evaluated with GraphPad Prism 6 (Graphpad Software, La Jolla, CA, USA). The results are presented as means ± standard deviation (SD). P values of < 0.05 were considered to be statistically significant.

## Results

### Optimal initial cell plating density

**Fig 2:**
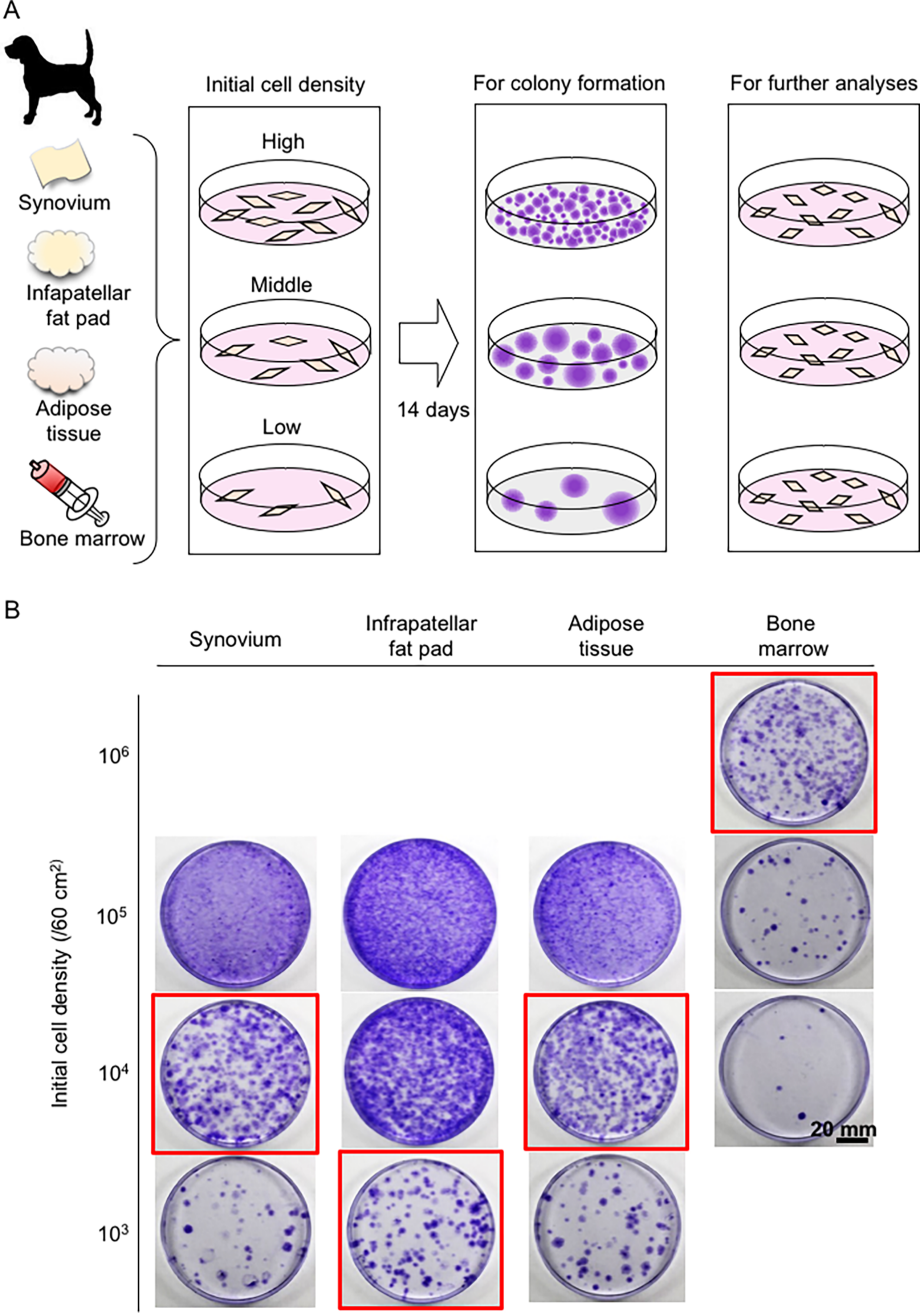
Optimal initial cell plating density at passage 0. (A) Experimental design. Nucleated cells derived from solid tissues were plated in six dishes at high, middle, and low density (1 ×10^3^, 1 ×10^4^, or 1 ×10^5^ cells per dish; 60-cm^2^ dish), while cells derived from bone marrow were plated at 1 ×10^4^, 1 ×10^5^, or 1 ×10^6^ cells per dish (60-cm^2^ dish). After culturing for 14 days, three dishes from each condition were stained with crystal violet and the optimal initial cell density was determined based on the following criteria: 1) the colony size was not affected by colony-to-colony contact inhibition and 2) the greatest number of colonies was obtained. The cells from the remaining three dishes were used for further analyses. (B) Cell colonies stained with crystal violet. The optimal initial cell density is indicated by a red square.

**Fig 3:**
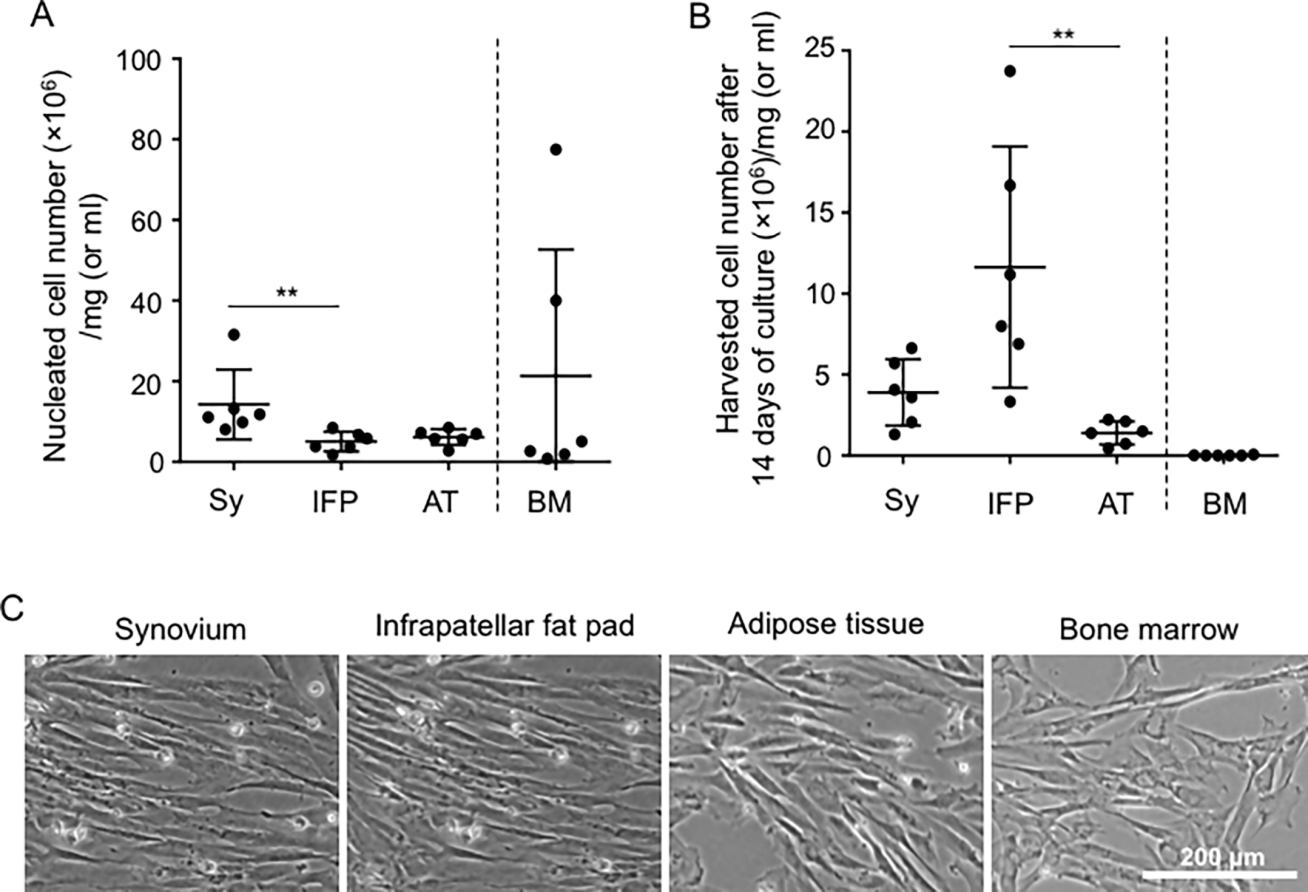
Nucleated cell number, harvested cell number, and cell morphology at passage 0. (A) The numbers of nucleated cells tissue weight and per bone marrow volume. Synovium, infrapatellar fat pad, and adipose tissues were digested with collagenase D solution, and nucleated cell numbers were counted for these three tissues as well as for bone marrow. (B) Harvested cell number after 14 days of culture. The nucleated cells were plated at the determined optimal plating density and after 14 days of culture, the numbers of harvested cells were counted. Values are the mean ± standard deviation (n = 6). Statistical analyses were carried out only for the three solid tissues. **, p < 0.01 with the Friedman test, followed by Dunn’s multiple comparisons test. Sy, synovium; IFP, infrapatellar fat pad; AT, adipose tissue; BM, bone marrow. (C) Cell morphology of plastic-adherent cells derived from four canine mesenchymal tissues.

Maximum yields of nucleated cells were obtained by plating nucleated cells derived from the four tissue types of the six canine donors at 3 different cell densities (Fig 2A). We found that the optimal initial cell density was 10^3^ cells/dish (60 cm^2^ dish) for the infrapatellar fat pad, 10^4^ cells/dish for synovium and adipose tissue, and 10^6^ cells/dish for bone marrow (Fig 2B). Comparison among the three solid tissues revealed that the nucleated cell numbers obtained from infrapatellar fat pad tissue (mg or ml) were lower than the numbers from synovium (Fig 3A), whereas the cell yields/tissue (mg or ml) for infrapatellar fat pad were greatly increased and significantly higher than those for adipose tissue at 14 days of culture (Fig 3B). Nucleated cells attached to plastic dishes were spindle shaped for all four canine tissues (Fig 3C).

### Surface marker expression

**Fig 4:**
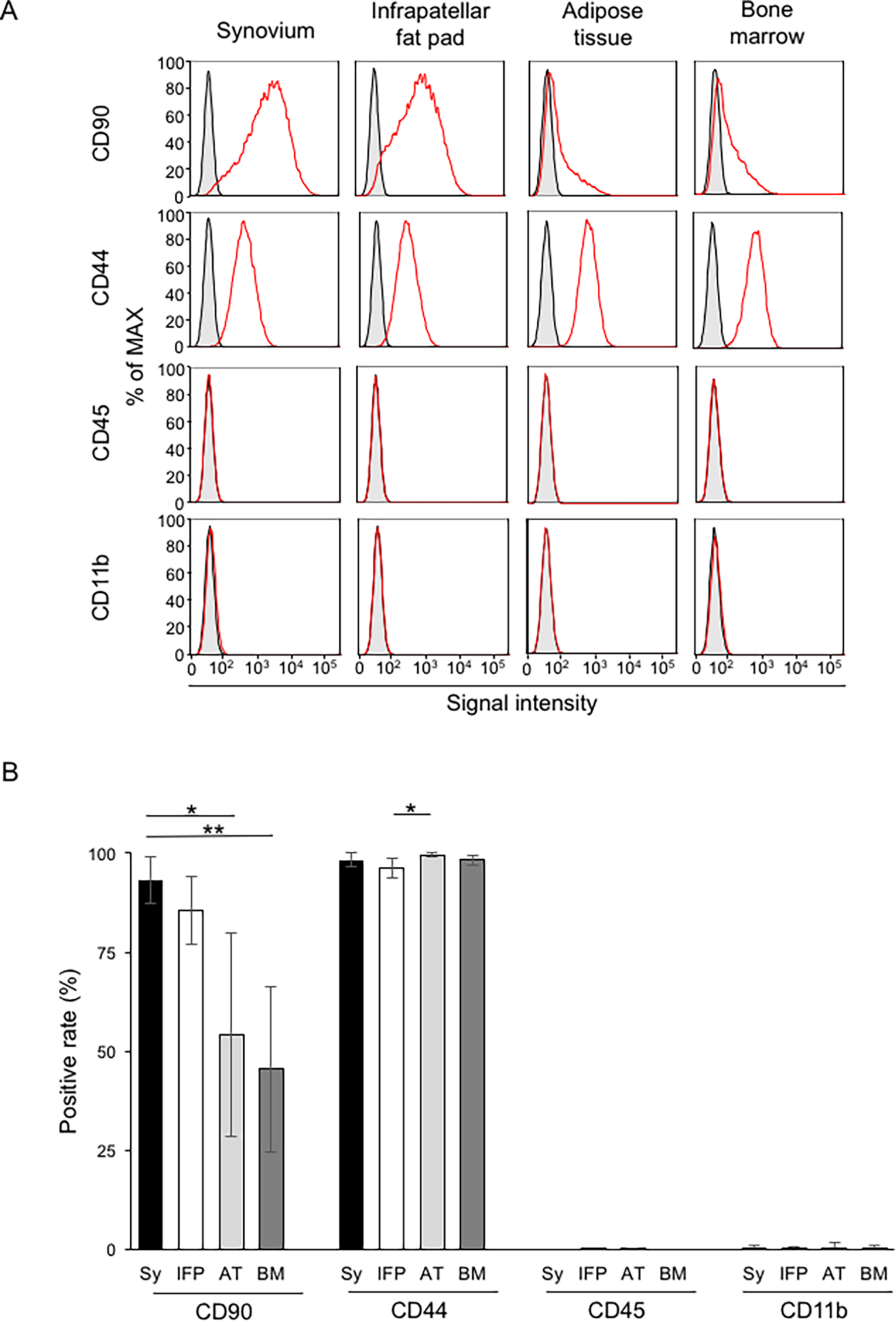
Surface marker expression of cells at passage 1. **(**A) Representative histograms of surface markers. Frozen cells at passage 0 were thawed, plated at 3 × 10^5^ cells/dish (150 cm^2^ dishes), and cultured for 7 days. Expressions of each surface marker are shown as red lines and for each isotype control as black lines. (B) Positive rate for each surface marker. Values are the mean ± SD (n = 6). *, p < 0.05. **, p < 0.01 with the Kruskal Wallis test, followed by Dunn’s multiple comparisons test. Sy, synovium; IFP, infrapatellar fat pad; AT, adipose tissue; BM, bone marrow.

Cells derived from all four tissue types were positive for CD90 and CD44 and negative for CD45 and CD11b (Fig 4A). The CD90 positive rate was significantly higher for synovium than for adipose tissue and bone marrow. The CD44 positive rate was over 96% for all tissues sources. (Fig 4B)

### Colony-forming ability and proliferation potential

**Fig 5:**
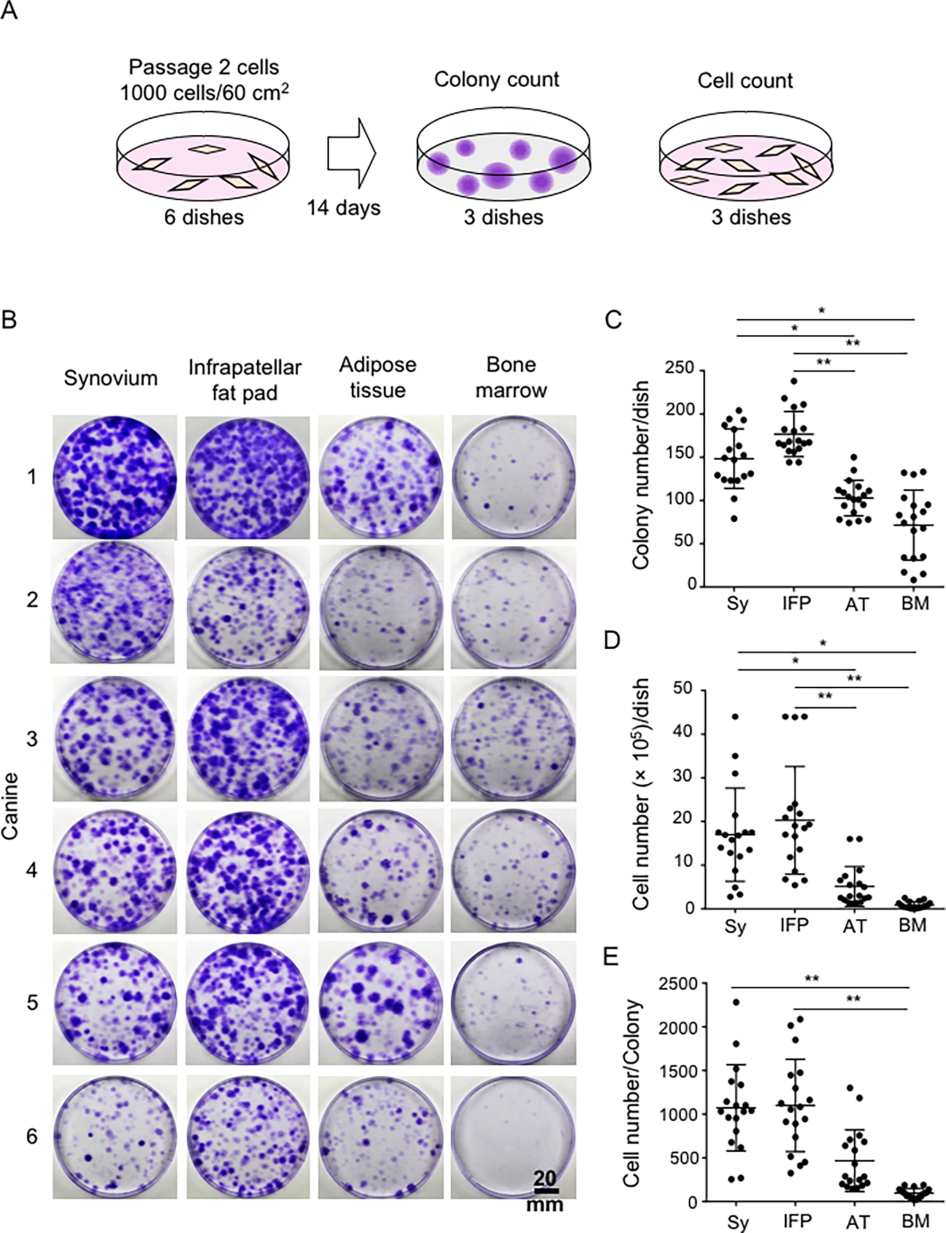
Colony forming ability of cells at passage 2. (A) Experimental design. Passage-2 cells were plated at 1 × 10^3^ cells/dish (60 cm^2^ dishes) in 6 dishes. After 14 days of culture, three dishes were stained with crystal violet for colony counting and the other three dishes were used for cell counting and further analyses. (B) Images of colonies stained with crystal violet. (C) Colony number (1 × 10^5^)/dish (60 cm^2^ dishes). (E) Cell number/colony. The means ± SD are shown (n = 18). *, p < 0.05. **, p < 0.01 with the Kruskal Wallis test, followed by Dunn’s multiple comparisons test.

Colony forming abilities were compared by plating passage-2 cells derived from the four tissues at 1 × 10^3^ cells in 60 cm^2^ dishes and culturing for 14 days (Fig 5A). The colony numbers per dish were higher for synovium and for the infrapatellar fat pad tissues than for adipose tissue or bone marrow (Fig 5B and 5C). The cell numbers per dish were also higher for the synovium and infrapatellar fat pad tissues than for adipose tissue and bone marrow (Fig 5D). The cell numbers per colony were higher for synovium and infrapatellar fat pad tissues than for bone marrow (Fig 5E).

**Fig 6:**
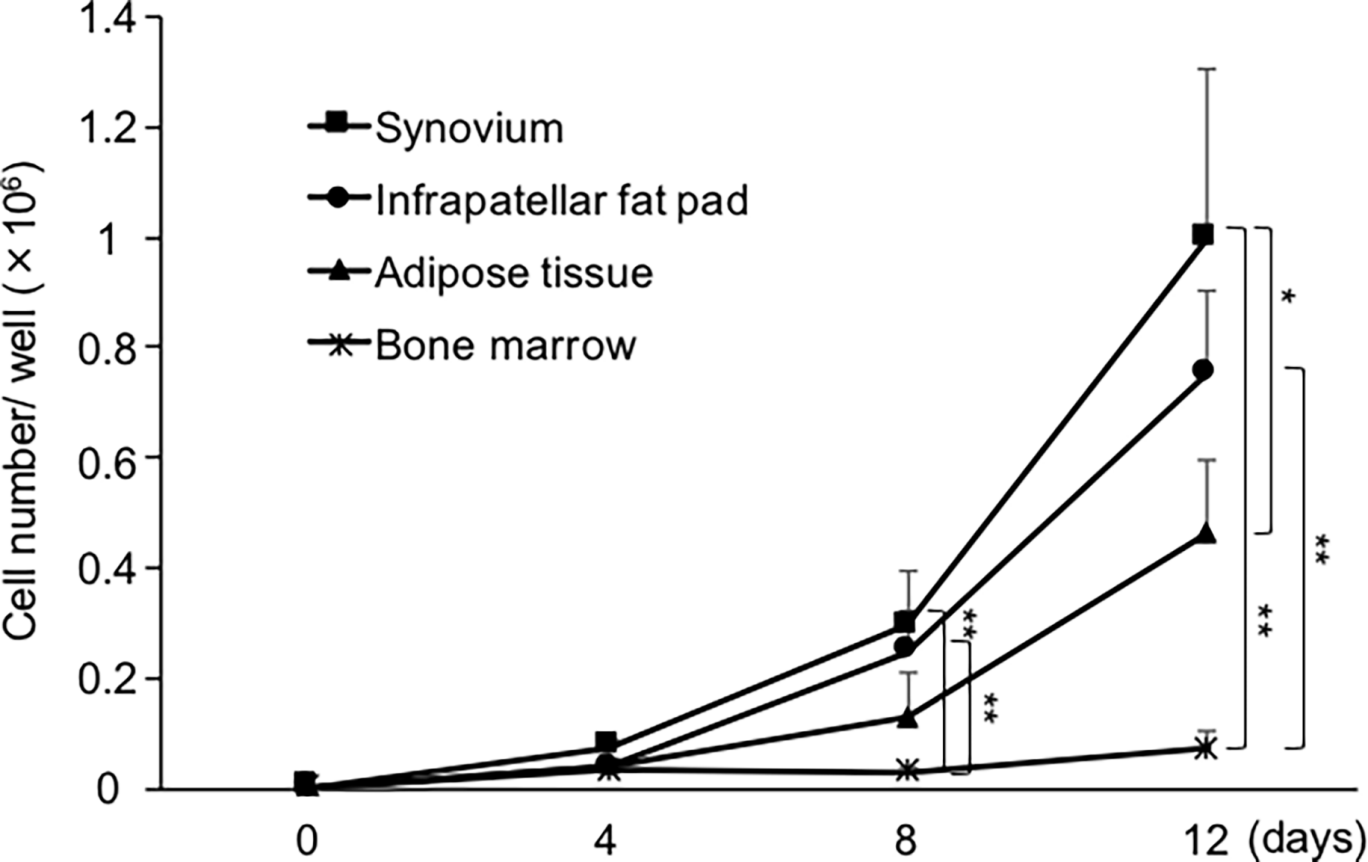
Proliferation potential of cells at passage 2. Cells were plated at 1 × 10^3^ cells/10 cm^2^ (3 canine donors, n = 3 for each donor) and cell numbers were counted after 4, 8, and 12 days of culture. The means ± SD are shown. *, p < 0.05. **, p < 0.01 with the Friedman test, followed by Dunn’s multiple comparisons test.

Proliferation potential was compared by plating passage-2 cells derived from the four tissues at 1 × 10^3^ cells/10 cm^2^. After 8 and 12 days of culture, the numbers of proliferated cells were significantly greater for synovium and infrapatellar fat pad tissues than for bone marrow. At 12 days of culture, the numbers of proliferated cells were greater for synovium than for adipose tissue (Fig 6).

### Chondrogenesis

**Fig 7:**
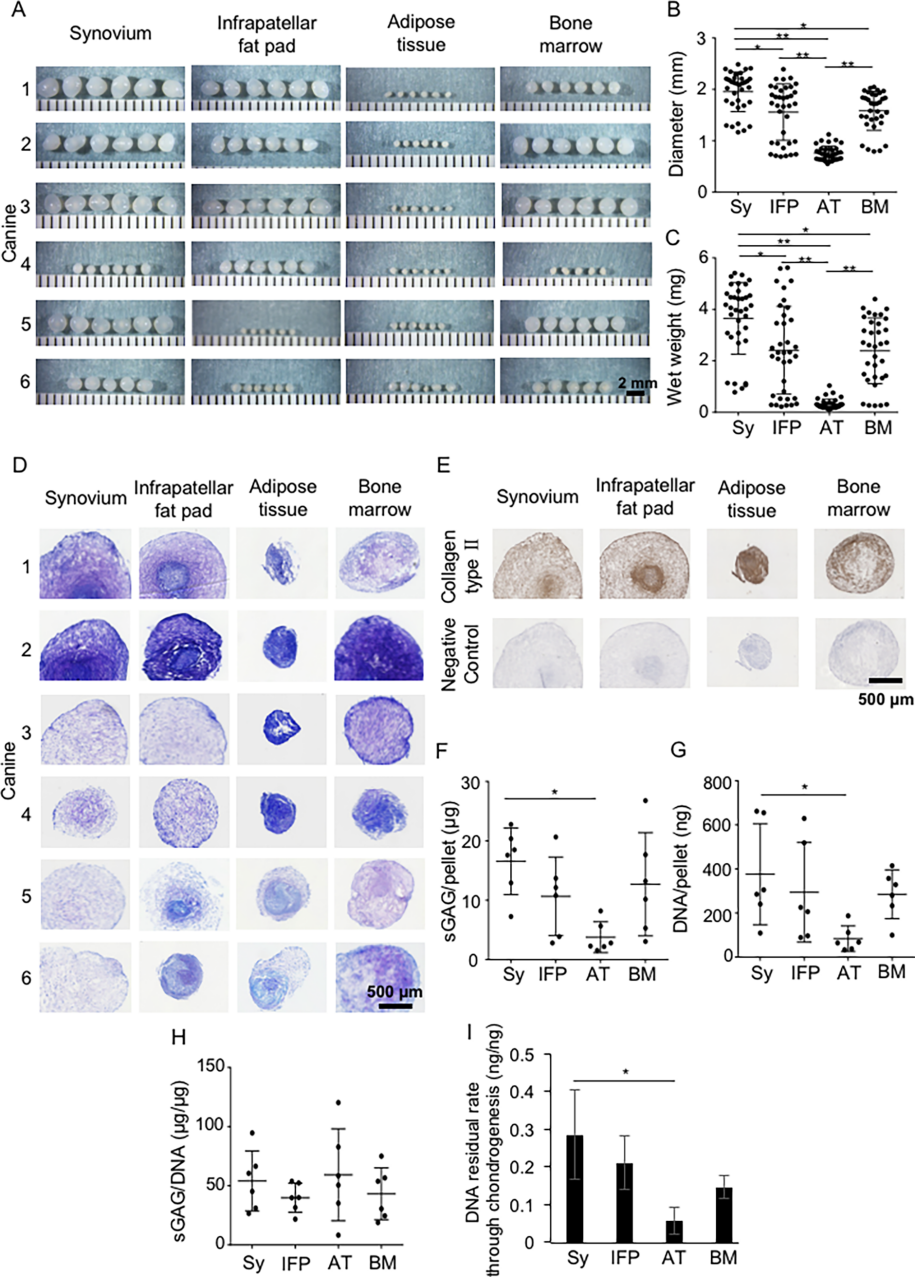
Chondrogenic potential of cells at passage 2. An aliquot containing 2.5 × 10^5^ passage-2 cells was pelleted and cultured in chondrogenic differentiation medium for 21 days. (A) Macroscopic photographs of cartilage pellets (6 pellets per canine; 6 canine donors). (B) Diameter of cartilage pellets (n = 36). (C) Wet weight of cartilage pellets (n = 36). (D) Histology of sections stained with toluidine blue. (E) Histology of sections immunostained for collagen type II. (F) Amount of sulfated glycosaminoglycan (sGAG) per pellet (n = 36). (G) DNA amount per pellet (n = 36). (H) Ratio of sGAG amount/DNA amount (n = 36). (I) DNA residual rate during chondrogenesis (= DNA amount at 21 days/DNA amount at 0 day; 3 pellets per canine; 3 canine donors). The means ± SD are shown. *, p < 0.05. **, p < 0.01 with the Kruskal Wallis test, followed by Dunn’s multiple comparisons test.

After chondrogenic differentiation, each cartilage pellet was translucent, but the size and wet weight varied depending on the donor and the type of mesenchymal tissue harvested (Fig 7A). Control pellets cultured without BMP-2 and TGFβ were too small to perform further detailed analyses; however, no significant differences were observed among the four tissues (S1 Fig). Pellets cultured in chondrogenic medium showed the largest diameters and wet weights for synovium and the smallest for adipose tissue (Fig 7B and 7C). All pellets were stained with toluidine blue (Fig 7D) and immunostained for collagen type II (Fig 7E). The staining intensity of toluidine blue and levels of immunostaining for collagen type II did not differ among the four cell types. The amounts of sGAG and DNA per pellet were higher for synovium than for adipose tissue (Fig 7F and 7G). The sGAG/DNA ratio was equivalent in each tissue (Fig 7H). The DNA residual rate throughout chondrogenesis was higher for synovium than for adipose tissue (Fig 7I).

### Calcification

**Fig 8:**
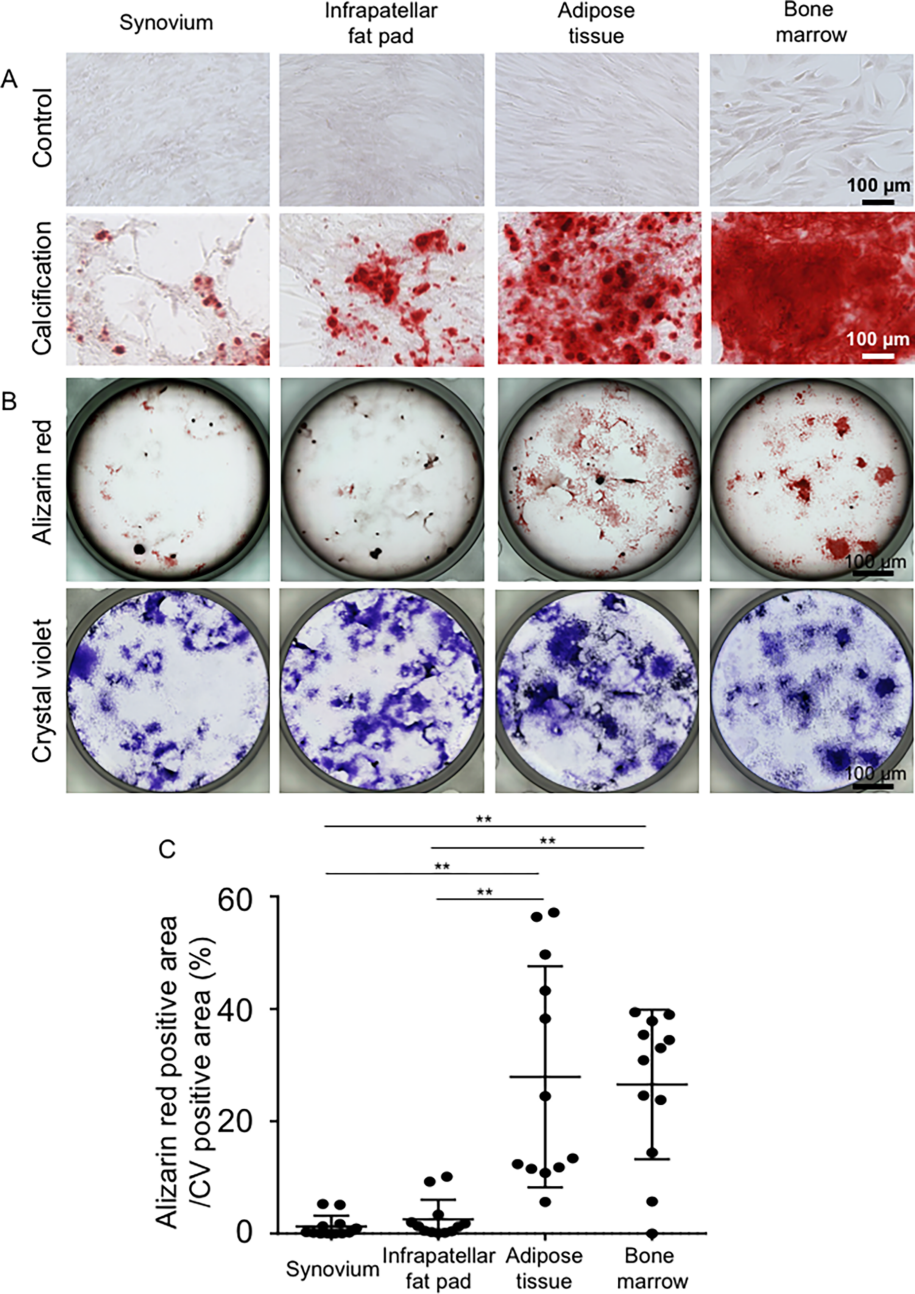
Calcification potential. (A) Cells cultured with and without calcification medium. Passage-3 cells were plated at 1 × 10^3^ cells/10 cm^2^ well, precultured for 7 days, and then cultured with or without calcification induction medium for 21 days. (B) Alizarin red positive colonies and total colonies. The wells were first stained with alizarin red, and then the same wells were stained with crystal violet. (C) Ratio (%) of alizarin red positive/crystal violet positive areas. The means ± SD are shown. *, p < 0.05. **, p < 0.01 with the Kruskal Wallis test, followed by Dunn’s multiple comparisons test.

After calcification, the cells from each mesenchymal tissue were stained with alizarin red (Fig 8A). Cells from all four tissues sources showed no staining with alizarin red when cultured in control medium. During calcification, canine MSCs were easily detached from culture dishes, irrespective of their sources. Ratios of alizarin red positive area/CV positive area ratios were higher for cells derived from adipose tissue and bone marrow-derived cells than for cells derived from synovium and infrapatellar fat pads (Fig 8B and 8C).

### Adipogenesis

**Fig 9:**
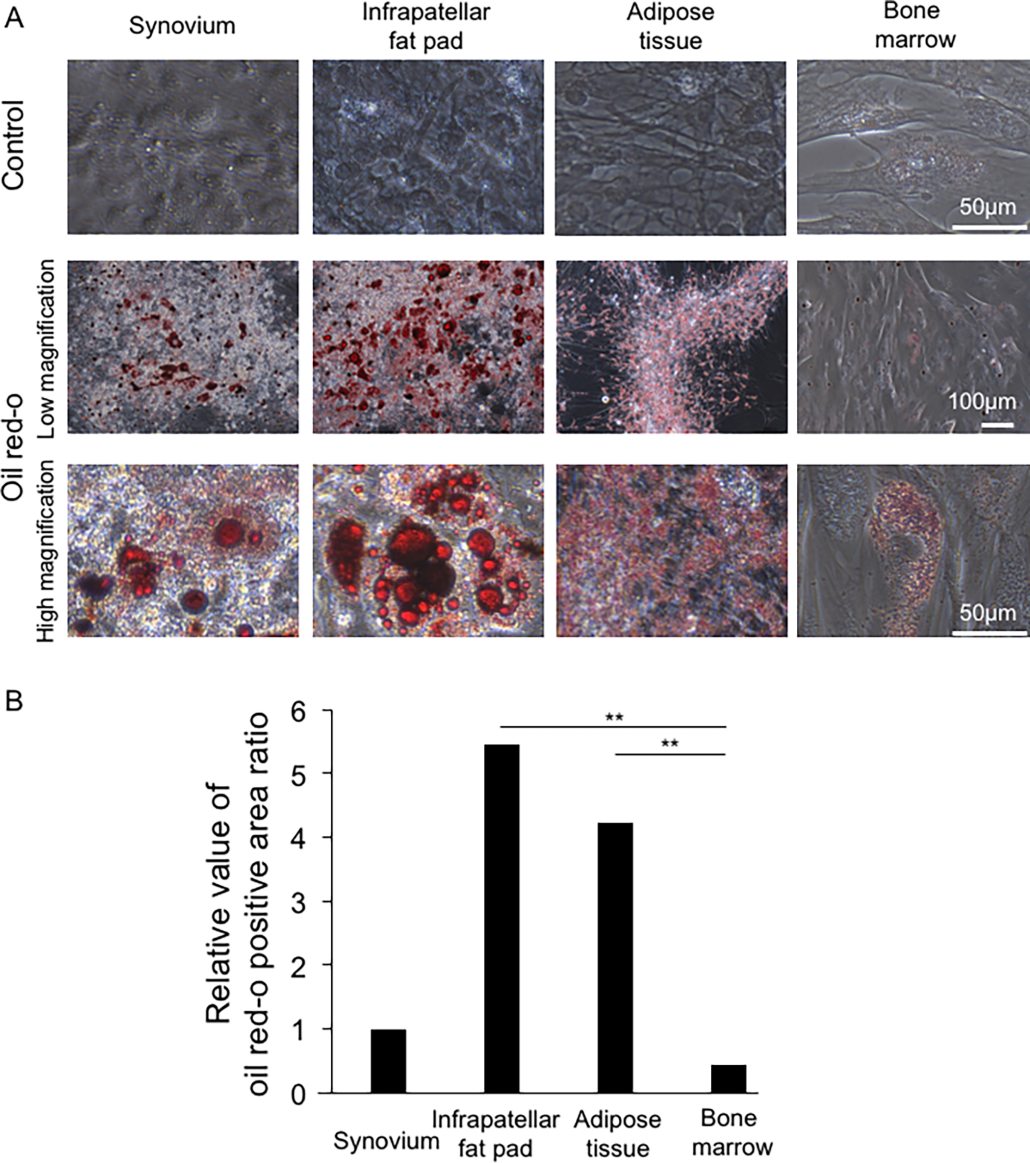
Adipogenic differentiation potential. (A) Cells cultured with and without adipogenic medium. Passage-3 cells were plated at 2 × 10^4^ cells/5 cm^2^ well, precultured for 7 days, then cultured with or without adipogenic differentiation medium for 21 days. (B) Relative values of the ratios of the oil red-o positive areas against the ratios for synovium-derived cells. The means ± SD are shown. *, p < 0.05. **, p < 0.01 with the Kruskal Wallis test, followed by Dunn’s multiple comparisons test.

Control cultures showed no droplets that stained with oil red-o. After adipogenesis with adipogenic differentiation medium, the cells derived from four canine tissue sources produced lipid droplets that stained with oil red-o (Fig 9A). Large lipid droplets were observed for synovium- and infrapatellar fat pad-derived cells. In contrast, small lipid droplets were observed for adipose tissue- and bone marrow-derived cells. Ratios of oil red o positive areas were higher for cells derived from infrapatellar fat pad and adipose tissue than from bone marrow (Fig 9B).

## Discussion

A precise definition for the term MSC remains confusing, but the minimal criteria for human MSCs, as advocated by the International Society for Cellular Therapy (ISCT), are that the cells (1) are plastic-adherent under standard culture conditions; (2) express CD105, CD73, and CD90, but lack expression of CD45, CD34, CD14 or CD11b, CD79a or CD19, and HLA-DR surface molecules; and (3) differentiate into osteoblasts, adipocytes, and chondrocytes [20]. We were unable to examine the full complement of surface markers recommended by ISCT because of a lack of appropriate canine antibodies, but the colony-forming cells we isolated from canine synovium, infrapatellar fat pad, adipose, and bone marrow tissues all met the criteria listed above, indicating that the cells were canine MSCs.

In this study, we used donor-matched samples for comparisons because of the known occurrence of variations in the properties of MSCs among individuals [12, 21, 22]. The MSCs were also compared at the same passage, because passage number can also affect the properties of MSCs [23–26]. For the primary culture, nucleated cells were plated at a density that would avoid interactions between cell colonies and promote expansion at a maximum rate, as colony-to-colony contact can affect the cell size, surface epitopes, proliferation potential, and differentiation potential [12, 13, 27–29]. We determined the optimal initial cell density based on the following criteria: (1) the colony size was not affected by colony-to-colony contact inhibition and (2) the greatest number of colonies was obtained. The optimal initial cell density was 100-fold higher for cells from bone marrow than from synovium, infrapatellar fat pad, or adipose tissues (Fig 2). Similar results were reported for human [12, 27] and rat [13] MSC sources. The MSC yields at passage 0, after 14 days of incubation, were the highest for the infrapatellar fat pad tissues (Fig 3). Theoretically, the total cell yield at passage 0 is determined by the “harvested sample weight or volume” multiplied by the “cell yields/tissue (mg or ml).” If the collection quantity is limited, the infrapatellar fat pad has an advantage of producing a high MSC yield at passage 0.

The canine MSCs from each tissue were positive for CD90 and CD44 and negative for CD45 and CD11b (Fig 4), in agreement with many MSC studies on humans [12, 27, 30] and canines [21, 31–33]. The CD44 positive rate was over 96% in each tissue source, but the CD90 positive rate depended on the tissue source. Many studies in humans and rats have reported robust expression of CD90 [12, 13, 34], whereas some mouse and canine studies have shown low expression of CD90 [31, 35, 36]; thus, the CD90 positive rate of MSCs differs among species or tissue sources. The present results support a relationship between the CD90 positive rate and chondrogenic potential, as previously reported in humans [37] and canines [37, 38]. We demonstrated a higher colony number and cell number/colony for MSCs at passage-2 when derived from the synovium and infrapatellar fat pad rather than from adipose tissue or bone marrow (Fig 5). The MSCs derived from the synovium and infrapatellar fat pad also demonstrated higher proliferation capacity (Fig 6). A higher proliferation potential has also been reported for human MSCs derived from synovium rather than from adipose tissue or bone marrow [12, 17].

We quantitatively evaluated the chondrogenic differentiation potential of MSCs from each of the four tissue sources (Fig 7). Our previous study revealed that the pellet formed during *in vitro* chondrogenesis by MSCs increased in size and wet weight due to the production of extracellular matrix, whereas the cell number decreased [39]. In the present study, the diameters and wet weights of the cartilage pellets were the highest for the synovium. The sGAG/pellet, DNA amount/pellet, and DNA residual rate through chondrogenesis were higher for the synovium than for the adipose tissue. By contrast, no significant differences were observed for the sGAG/DNA ratio or the staining intensity for toluidine blue and collagen type II in all four sources, indicating a similar cartilage matrix production per cell in all four sources. These findings suggest that the differences in sGAG amounts per pellet among the four canine tissues were due to the differences in cell viability in the different cartilage pellets.

For calcification, we used a differentiation medium similar to that used for human MSCs. Unlike human MSCs, we found that canine MSCs were easily detached during calcification, irrespective of their sources, as previously reported for canine adipose tissue-derived stem cells [9]. In the present study, the calcification potential was higher for canine adipose tissue- and bone marrow-derived MSCs than for synovium- and infrapatellar fat pad-derived MSCs (Fig 8). These results differ from previous findings for humans or rats, where higher calcification potential was reported for synovium-derived MSCs than for MSCs derived from adipose tissue or bone marrow [12, 13]. These results indicate that some species specificity may exist for calcification.

We were unable to differentiate canine MSCs from each source into adipocytes using an adipogenesis differentiation medium designed for human MSCs [40]. We therefore switched to a canine-specific adipogenesis medium proposed by Neupane *et al*. [9], which contained rosiglitazone and insulin instead of isobutyl-methyl xanthine and indomethacin. This canine-specific adipogenesis medium worked well (Fig 9). In the present study, the adipogenic differentiation potential was higher for MSCs derived from the infrapatellar fat pad and adipose tissue than from bone marrow, whereas synovium-derived MSCs showed no superiority in terms of adipogenic differentiation potential. These results differed from those of previous studies on humans and rats, which showed that synovium- and infrapatellar fat pad-derived MSCs showed high adipogenic differentiation potential [12, 13, 17]. Nevertheless, the results from the present study are reasonable considering the characteristics of infrapatellar fat pad and adipose tissue, which consist mainly of fat.

In the present study, the synovium and infrapatellar fat pad were harvested from the knee, adipose tissue was harvested subcutaneously from the inguinal region, and bone marrow was aspirated from the proximal humerus (Fig 1). With regard to accessibility in a practical clinical situation, bone marrow has an advantage because it can be collected using only a bone marrow needle without a long incision. If an arthroscopy system is available, synovium and infrapatellar fat pad tissues can be harvested from the knee with two small incisions. In humans, adipose tissue can be easily harvested from the abdomen by liposuction, while in canines, adipose tissue retrieval can be the most invasive of the four procedures.

The currently available reports of canine MSC production typically have utilized adipose tissue and bone marrow [6, 8, 9, 31]. Both these sources have their own attractiveness, but the resulting MSCs are not particularly good for use in cartilage regenerative medicine because they do not show high proliferative and chondrogenic potential. However, MSCs can be isolated from a number of different adult mesenchymal tissues. Our previous comparison studies confirmed that MSCs derived from the synovium from rats [13], rabbits [14], pigs [16], and humans [12] all showed pronounced proliferative and chondrogenic properties. In the present study, we confirmed the superiority of the synovium as a cell source for cartilage regeneration therapy in canines, based on the high proliferation ability, abundant chondrogenic differentiation, and similar quality of regenerated cartilage for MSCs derived from synovium when compared to MSCs from adipose tissue and bone marrow. Thus, we propose the canine synovium as a source of MSCs for cartilage regenerative medicine. We previously demonstrated that the *in vitro* chondrogenic potential of MSCs reflected the *in vivo* chondrogenic potential in a rabbit cartilage defect model [14]; therefore, we expect that canine synovium-derived MSCs will also demonstrate high chondrogenic potential *in vivo*. The clinical safety and efficacy of these MSCs remains to be confirmed in canines in the *in vivo* situation.

## Conclusions

We expanded canine MSCs by conducting a similar and strictly controlled process, and then performed a donor-matched quantitative and qualitative comparison to clarify the properties of MSCs derived from synovium, infrapatellar fat pad, adipose tissue, and bone marrow. The colony formation rates of nucleated cells, positive rate for CD90, proliferation potential, and differentiation capacity of the MSCs depended on the source of mesenchymal tissue. The MSCs derived from synovium had superior chondrogenic ability and high proliferation potential, making the synovium suitable as a source for MSCs for cartilage regeneration in canines.

## Supporting information

S1 Fig Chondrogenesis without TGFβ and BMP-2. Macroscopic images of control pellets and pellets cultured in chondrogenic differentiation medium.
(TIFF)
