# Supplementary figures and images for: Canine mesenchymal stem cells from synovium have a higher chondrogenic potential than those from infrapatellar fat pad, adipose tissue, and bone marrow
Source: PLoS One. 2018 Aug 23;13(8):e0202922. doi: 10.1371/journal.pone.0202922 (PMC6107231; doi:10.1371/journal.pone.0202922)

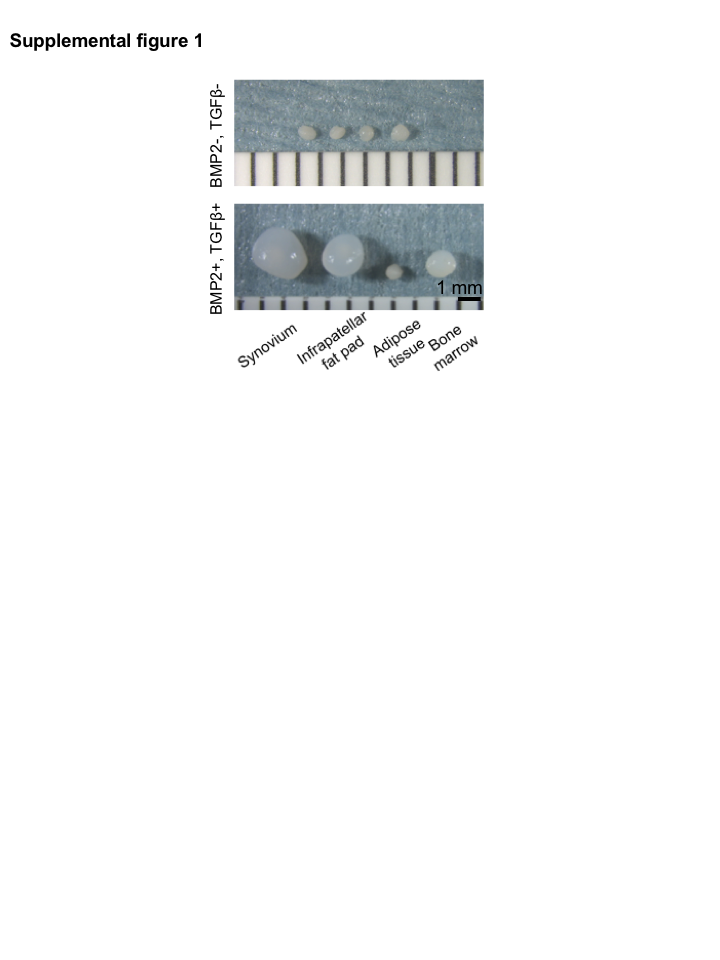

Supplement: S1 Fig — Macroscopic images of control pellets and pellets cultured in chondrogenic differentiation medium. (TIFF) [file pone.0202922.s001.tiff]
